# Mastitis-Associated Bacterial Isolates and Antimicrobial Resistance in a Single Dairy Herd in the Akmola Region, Kazakhstan

**DOI:** 10.3390/antibiotics15090889

**Published:** 2026-09-10

**Authors:** Assel Akhmetova, Ayan Dauletov, Alexander Ostrovskii, Nurdina Yerzhanova, Alexandr Shevtsov, Laura Dushayeva, Askar Nametov, Raushan Rychshanova, Asylulan Amirgazin, Marat Kuibagarov

**Affiliations:** 1 National Center for Biotechnology, Astana 010000, Kazakhstan; akhmet.assel@gmail.com (A.A.);; 2 Veterinary and Agrotechnology Institute, Zhangir Khan West Kazakhstan Agrarian Technical University, Uralsk 090009, Kazakhstan; 3 Scientific Research Institute of Innovative Technologies, Akhmet Baitursynuly Kostanay Regional University, Kostanay 110000, Kazakhstan

**Keywords:** bovine mastitis, antimicrobial resistance, susceptibility testing, mobile genetic elements, *Staphylococcus*, *Streptococcus*, *Escherichia coli*, *Aerococcus*, *Corynebacterium*

## Abstract

Background/Objectives: Bovine mastitis is a common infection in cattle causing significant economic losses globally in the dairy industry. While bacterial profiles and antimicrobial resistance (AMR) patterns are generally consistent worldwide, comprehensive epidemiological and AMR data from Kazakhstan are lacking. This study characterized mastitis-associated bacterial isolates and their AMR profiles from a single commercial Simmental dairy herd in the Akmola Region, Kazakhstan. Milk samples from 41 cows with clinical mastitis and documented treatment histories were collected. Methods: Bacterial identification was performed using MALDI-TOF MS, and antimicrobial susceptibility was tested phenotypically following EUCAST criteria. Whole genome sequencing (WGS) on a subset of isolates analyzed resistance and plasmid content using AMRFinderPlus v3.11.14 and Abricate v1.4.0, with the ResFinder and PlasmidFinder databases, respectively. Results: All samples yielded bacterial growth, resulting in 58 isolates across 11 species and five genera, from which *Staphylococci* predominated (50.0%), followed by *Streptococci* (28.0%), *Escherichia coli* (19%), one *Aerococcus* (1.72%) and one *Corynebacterium* (1.72%) strain. Phenotypic resistance to at least one antimicrobial class was found in 65.5% of tested isolates. Tetracycline resistance was the most frequent (71.7% of tested isolates), with multidrug resistance detected in 6.9%. WGS of a randomly selected subset of 33 isolates revealed that 27 (82%) carried at least one acquired AMR gene, predominantly tetracycline resistance genes (26/33), followed by lincosamide/macrolide (12 and 11 isolates) and aminoglycoside resistance genes (9 isolates). Plasmid replicon screening identified plasmid replicons in 21 of the 33 sequenced isolates, mainly of the repUS43 and repUS76 staphylococcal replicon families, suggesting a potential role of plasmids in AMR gene dissemination within the herd. Conclusions: This study offers a comprehensive phenotypic and genomic characterization of mastitis-associated pathogens and their AMR determinants within this single herd, highlighting the co-circulation of tetracycline, lincosamide/macrolide, and aminoglycoside resistance genes. As this study was limited to a single dairy herd, these findings should be regarded as descriptive, farm-level observations rather than being representative of the Akmola Region or Kazakhstan more broadly. These findings support farm-level mastitis surveillance that combines species identification, phenotypic AMR testing, and targeted molecular screening.

## 1. Introduction

Bovine mastitis remains one of the most important infectious and inflammatory diseases in dairy cattle. As one of the most common cattle diseases globally, it continues to cause substantial losses in both animal health and the economic sustainability of dairy production [1,2]. This inflammation of the mammary gland drastically reduces milk yield and quality [3,4,5]. Consequently, despite ongoing preventive and therapeutic measures, managing mastitis remains a primary challenge for the dairy industry worldwide [6].

The etiology of bovine mastitis is highly heterogeneous and includes various Gram-positive and Gram-negative bacteria [7]. Traditionally, the most clinically relevant pathogens belong to the genera *Staphylococcus*, *Streptococcus*, and *Escherichia*. However, these bacterial groups may differ in pathogenicity, ecological niche, transmission routes, epidemiological behavior, and antimicrobial susceptibility profiles [8,9]. Recent studies increasingly recognize non-*aureus* staphylococci (NAS) and selected coryneform bacteria as relevant mastitis-associated organisms, although their clinical significance may vary by species and herd context [10,11]. Variation in mastitis-associated bacterial communities highly depends on herd management practices, especially those related to hygiene, environmental exposure, and production system characteristics [12].

The widespread use of antimicrobial agents is another challenge in mastitis control. In everyday practice on dairy farms, therapeutic decisions are often made before laboratory confirmation of the causative agent, which may contribute to the selection of resistant bacterial populations and compromise treatment efficacy [9,13]. In recent years, antimicrobial resistance (AMR) among mastitis-associated bacteria has become a growing concern not only in veterinary medicine but also within the One Health framework, as the mammary gland and milk can serve as reservoirs of resistant microorganisms and antimicrobial resistance genes [14,15]. The circulation of mobile genetic elements is particularly concerning, as they can mediate the horizontal transfer of resistance determinants among taxonomically diverse bacterial groups, thereby fostering the maintenance and spread of AMR in dairy production systems [14,16].

Accordingly, contemporary investigations of bovine mastitis require a comprehensive laboratory approach that extends beyond conventional bacteriological culture. While culture-based diagnostics remain essential for pathogen recovery, advanced analytical methods are increasingly necessary to accurately characterize the etiological diversity and resistance profiles of mastitis-associated bacteria. The use of MALDI-TOF mass spectrometry substantially improves the accuracy and speed of bacterial identification, while phenotypic antimicrobial susceptibility testing provides clinically relevant information for treatment selection [17,18]. In parallel, molecular screening of selected antimicrobial resistance genes and mobile-element markers can complement phenotypic testing by indicating the presence of resistance-associated genetic determinants [19,20]. Whole-genome sequencing further extends this molecular characterization beyond targeted marker screening, enabling comprehensive detection of the full repertoire of acquired resistance genes and associated plasmid replicons within a single assay, thereby providing a more complete picture of the genetic basis of resistance and its potential for mobility [21,22].

Because the etiological structure of mastitis and the resistance profiles of associated bacterial isolates may differ markedly across countries, regions, farms, and management systems, local microbiological surveillance remains essential for evidence-based therapeutic decision-making and optimization of mastitis control strategies [9,12]. Such data are particularly valuable in herd-level settings where empirical antimicrobial treatment is used, and local microbiological evidence is limited. However, data on species-level mastitis-associated bacterial isolates and their phenotypic and molecular AMR profiles in Kazakhstan herds remain limited.

Therefore, this study aimed to characterize bacterial pathogens associated with bovine mastitis and their antimicrobial resistance profiles in a single commercial dairy herd using phenotypic and genomic approaches. Although whole-genome sequencing enabled comprehensive characterization of selected isolates, the findings should be interpreted as herd-specific observations and not as representative of the broader dairy cattle population in Kazakhstan.

## 2. Results

### 2.1. Bacteriological Examination of Milk Samples

Bacteriological analysis was performed for 41 milk samples collected from cows with clinical signs of mastitis. From them, 15 samples were obtained from animals 48 h after two doses of cefquinome, and 26 samples from animals without prior antibiotic treatment. One milk sample per animal was obtained from one clinically affected udder quarter for analysis.

Bacterial growth was detected in all examined samples. A total of 58 bacterial isolates were recovered, representing 11 species and 5 genera: *Staphylococcus*, *Streptococcus*, *Escherichia*, *Aerococcus* and *Corynebacterium* (Table 1).

Of the 58 bacterial isolates recovered, *Staphylococcus* spp. were the most prevalent (29 isolates; 50.0% of all isolates), followed by *Streptococcus* spp. (16 isolates; 27.6%), *Escherichia coli* (11 isolates; 19.0%), *Aerococcus viridans* (1 isolate; 1.7%) and *Corynebacterium* stationis (1 isolate; 1.7%).

At the isolate level (*n* = 58), the most frequently recovered species were *Staphylococcus aureus* (*n* = 11; 19.0% of all isolates), *Streptococcus dysgalactiae* (*n* = 8; 13.8%), *Streptococcus uberis* (8; 13.8%), *Escherichia coli* (*n* = 11; 19.0%), *Staphylococcus chromogenes* (*n* = 7; 12.1%), *Staphylococcus hyicus* (*n* = 7; 12.1%) and *Staphylococcus simulans* (*n* = 2; 3.4%). *Staphylococcus epidermidis*, *Staphylococcus haemolyticus*, *Aerococcus viridans* and *Corynebacterium* stationis were each represented by a single isolate (1.7%).

A notable difference in pathogen distribution was observed between the treatment groups when expressed on a cow basis. Among the 15 cefquinome-pretreated cows, *S. aureus* was recovered from 11 animals (73.3% of the treated group), whereas it was not detected in any of the 26 untreated cows. Conversely, *Streptococcus* spp. predominated in the untreated group, recovering from 16 of the 26 untreated cows (61.5%), compared with a smaller proportion of the treated cohort. *Corynebacterium* stationis and *Aerococcus viridans* were identified only in untreated cows, whereas *E. coli* was detected in both groups (3 of 15 treated cows, 20.0%; 8 of 26 untreated cows, 30.8%).

### 2.2. Phenotypic Antimicrobial Susceptibility

Among the 58 phenotypically characterized isolates, 38 (65.5%) were resistant to at least one antimicrobial agent for which an applicable EUCAST interpretive criterion was available. Resistance frequency varied among bacterial species: all *Streptococcus uberis* (8/8) and *Streptococcus dysgalactiae* (8/8) isolates were resistant to at least one antimicrobial agent, followed by *Staphylococcus aureus* (8/11, 72.7%), *Staphylococcus chromogenes* (5/7, 71.4%), *Staphylococcus hyicus* (3/7, 42.9%), and *Escherichia coli* (3/11, 27.3%). Detailed disk diffusion results, including inhibition zone diameters and susceptibility interpretations for all 58 bacterial isolates, are provided in Supplementary Table S1.

Tetracycline resistance was the most common phenotype, detected in 33 of 46 isolates tested against this agent (71.7%), and was particularly prevalent among *S. uberis* (8/8, 100%), *S. dysgalactiae* (7/8, 87.5%), *S. aureus* (8/11, 72.7%), and *S. chromogenes* (5/7, 71.4%). Clindamycin resistance was the second most frequent phenotype (10/17, 58.8%) and occurred predominantly among streptococci, including 7/8 *S. uberis* and 2/8 *S. dysgalactiae* isolates. Lower resistance frequencies were observed for ampicillin (4/27, 14.8%), erythromycin (6/45, 13.3%), trimethoprim/sulfamethoxazole (4/40, 10.0%), ceftriaxone (1/11, 9.1%), ciprofloxacin (1/12, 8.3%), penicillin (1/28, 3.6%), cefotaxime (1/16, 6.2%), imipenem (2/40, 5.0%), gentamicin (1/40, 2.5%), and cefoxitin (1/40, 2.5%). No resistance to amoxicillin/clavulanic acid, tobramycin, meropenem, ofloxacin, norfloxacin, moxifloxacin, or vancomycin was detected among isolates for which applicable interpretive criteria were available.

Multidrug resistance, defined as resistance to antimicrobial agents from three or more classes, was identified in 4 of 58 isolates (6.9%): *Staphylococcus chromogenes* P71-C8, *Staphylococcus epidermidis* P71-E8, *Streptococcus uberis* P71-H6, and *Corynebacterium stationis* P71-C1. Isolate P71-H6 (*S. uberis*) exhibited resistance to penicillin, ampicillin, cefotaxime, erythromycin, clindamycin, and tetracycline, representing the broadest resistance phenotype observed in the collection.

### 2.3. Genomic Characterization of Antimicrobial Resistance

Genome assembly quality across the 33 isolates was generally consistent with short-read draft assemblies of bacterial genomes (Supplementary Table S2). Total assembly length ranged from 2.02 Mb (*Aerococcus viridans*, P71_D3_I163) to 5.38 Mb (*Escherichia coli*, P71_G1_I108), with GC content varying by genus as expected (*Staphylococcus* 32.1–37.2%, *Streptococcus* 36.4–39.5%, *E. coli* 49.6–50.5%, *Corynebacterium stationis* 54.8%, *Aerococcus* 39.6%). Contiguity varied considerably: contig counts ranged from 30 (P71_F4_I161, N50 = 573,940 bp) to 528 (P71_C7_I136, N50 = 9635 bp), with a median N50 of ~140 kb across the collection. All 33 assemblies were gap-free (0 N’s per 100 kbp). Two isolates—P71_C7_I136 (*S. dysgalactiae*) and P71_G1_I108 (*E. coli*)—stood out as notably more fragmented (>500 contigs, N50 < 116 kb) and should be interpreted with more caution in downstream gene-presence analyses, since fragmented assemblies can split or truncate genes across contig boundaries.

**Figure 1:**
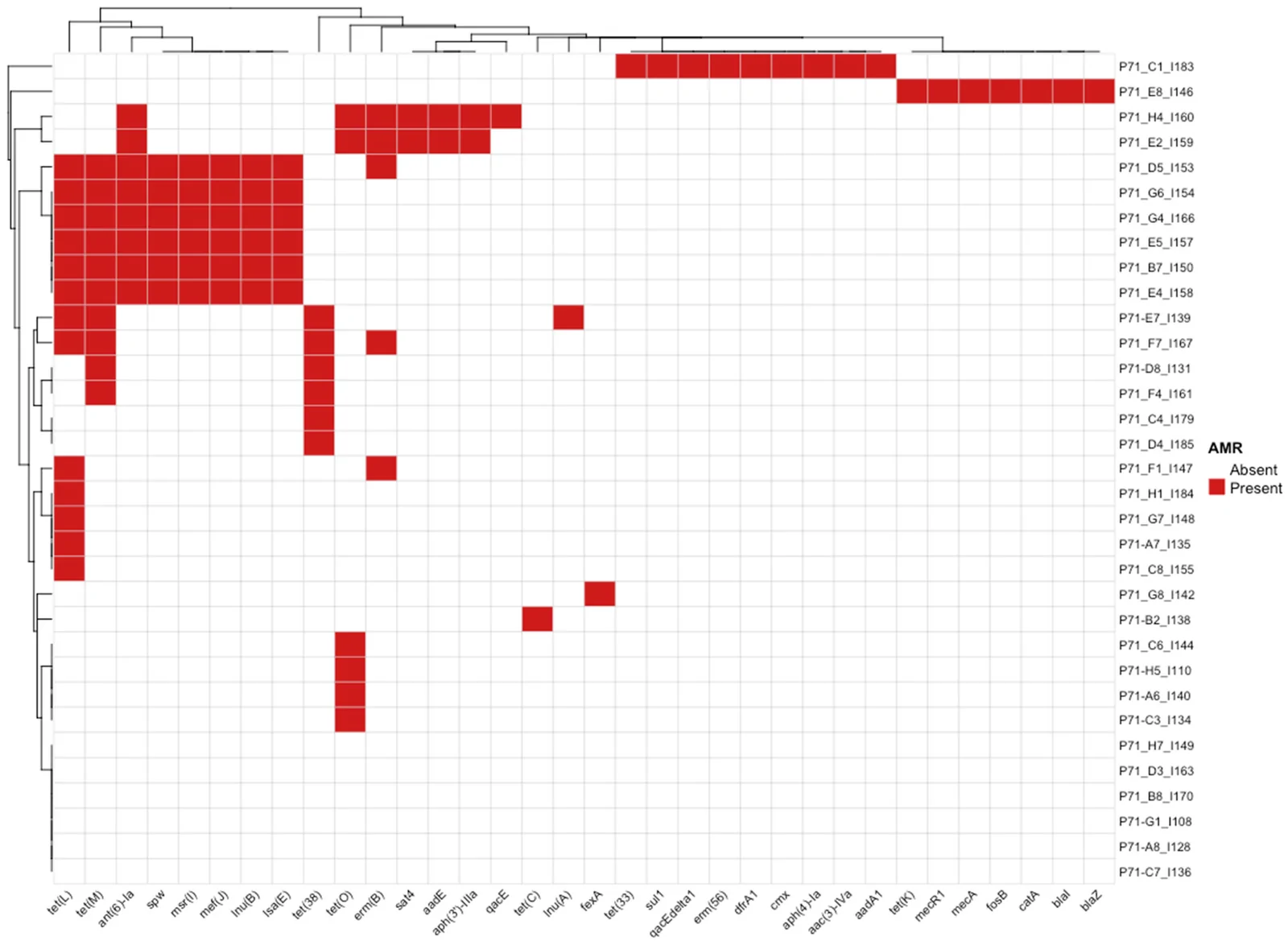
Heatmap showing the AMRFinderPlus results for 33 bacterial isolates screened for acquired antimicrobial resistance (AMR) genes.

Whole-genome sequences from 33 bacterial isolates were screened for acquired antimicrobial resistance (AMR) genes using AMRFinderPlus (Figure 1). Species identifications were dominated by *Streptococcus dysgalactiae* (*n* = 6), *Streptococcus uberis* (*n* = 6), *Staphylococcus aureus* (*n* = 6), *Staphylococcus chromogenes* (*n* = 5), *Staphylococcus hyicus* (*n* = 3), *Escherichia coli* (*n* = 2), and single representatives of *Staphylococcus simulans*, *Staphylococcus epidermidis*, *Staphylococcus haemolyticus*, *Corynebacterium stationis*, and *Aerococcus viridans*.

ResFinder was run for all 33 sequenced isolates. Output matched to 27 of these 33 isolates (81.8%); for the remaining 6 isolates (18.2%), ResFinder completed successfully but returned an empty output file, indicating no acquired resistance genes were detected above the default identity/coverage thresholds. These 6 isolates were therefore excluded from the tool-comparison analysis, although their AMRFinderPlus calls are reported separately. The 6 isolates with empty ResFinder output spanned six species (*S. hyicus*, *S. simulans*, *S. chromogenes*, *S. dysgalactiae*, *E. coli*, and *Aerococcus viridans*, one each) and did not skew the comparison toward any single taxon. Notably, all 6 of these isolates—P71_A8_I128 (*S. hyicus*), P71_B8_I170 (*S. simulans*), P71_C7_I136 (*S. dysgalactiae*), P71_D3_I163 (*Aerococcus viridans*), P71_G1_I108 (*E. coli*), and P71_H7_I149 (*S. chromogenes*)—likewise returned no AMRFinderPlus-detected acquired resistance genes, so their exclusion from the ResFinder comparison had no effect on the gene-level concordance statistics reported below.

Across the 27 isolates, a total of 102 unique isolate-gene calls were identified between the two tools combined (Supplementary Table S3). Gene nomenclature was harmonized prior to comparison, since three gene pairs were found to represent the same underlying gene reported under different naming conventions by the two curated databases: *aadA1* (AMRFinderPlus) and *ant(3″)-Ia* (ResFinder); *aph(3′)-IIIa* (AMRFinderPlus) and *aph(3′)-III* (ResFinder, which omits the terminal allele letter); and *catA* (AMRFinderPlus) and *cat(pC221)* (ResFinder, which reports the specific staphylococcal plasmid-borne allele name rather than the generic gene family designation). After comparison, 65 of the 102 gene calls (64%) were concordant between AMRFinderPlus and ResFinder, and no genes were detected by ResFinder that were not also detected by AMRFinderPlus.

The remaining 37 gene calls (36%) were identified by AMRFinderPlus alone (Table 2).

By contrast, the 65 concordant calls were dominated by well-characterised, horizontally mobile resistance genes with long-standing entries in both databases: *tet(L)* (11), *tet(M)* (10), *ant(6)-Ia* (8), *tet(O)* (6), *lnu(B)* (6), *lsa(E)* (6), plus single or double occurrences of *aac(3)-IVa*, *aadA1*, *aph(4)-Ia*, *cmx*, *dfrA1*, *sul1*, *tet(33)*, *lnu(A)*, *blaZ*, *catA*, *mecA*, *tet(K)*, *fexA*, *tet(C)*, and *aph(3′)-IIIa*.

A notable pattern is that six *Streptococcus uberis* isolates (P71_B7_I150, P71_D5_I153, P71_E4_I158, P71_E5_I157, P71_G4_I166, P71_G6_I154) each carried the identical cluster *mef(J)–msr(I)–spw*, detected by AMRFinderPlus but not by ResFinder in any of them, even though the co-located *lnu(B)* and *lsa(E)* genes on the same contigs were correctly detected by both tools in every one of these isolates. MLST performed for the six *S. uberis* isolates carrying the identical mef(J)–msr(I)–spw resistance-gene cluster (P71_B7_I150, P71_D5_I153, P71_E4_I158, P71_E5_I157, P71_G4_I166, P71_G6_I154) revealed an identical seven-locus allelic profile (arcC-1, ddl-1, gki-10, recP-1, tdk-2, tpi-1, yqiL-3) in all six isolates, which did not correspond to a currently defined sequence type in the PubMLST database.

The lowest agreement was seen in *S. aureus* (50.0%) and *S. epidermidis* (57.1%), driven by *tet(38)*, *erm(B)*, and the *mecA/blaZ* regulatory genes *mecR1* and *blaI*. The two streptococcal species, which together contributed 65 of the 102 total calls, sat in the middle of the range (56–61%), driven almost entirely by the recurring *mef(J)*/*msr(I)*/*spw* cluster. The three coagulase-negative/coagulase-variable staphylococci with small resistance gene counts (*S. chromogenes*, *S. hyicus*, *S. haemolyticus*) and the single *E. coli* isolate showed perfect (100%) concordance, though each contributed only 1–4 total calls, so these figures should be read with the small sample sizes in mind.

## 3. Discussion

Clinical mastitis in this Kazakhstani Simmental herd was dominated by staphylococci, characterized by frequent tetracycline resistance and a low prevalence of multidrug-resistant (MDR) phenotypes. Staphylococci accounted for a substantial proportion of all recovered isolates, with *Staphylococcus aureus* being one of the predominant species. This finding is consistent with published mastitis studies showing that staphylococci, including *S. aureus* and non-*aureus* staphylococci (NAS), as well as *Streptococcus* spp. and coliform bacteria, remain major bacterial groups associated with bovine mastitis [7,23,24,25,26]. In the present study, *Streptococcus dysgalactiae* and *Streptococcus uberis* were also frequently detected. NAS, particularly *S. chromogenes* and *S. hyicus*, were additionally recovered, supporting the need for species-level identification rather than genus-level reporting alone [27,28]. Phenotypic susceptibility testing revealed that tetracycline resistance was the predominant resistance profile, observed in 71.7% (33 of 46) of the isolates tested against this agent, whereas multidrug resistance (MDR) was identified in only 6.9% (4 of 58) of bacterial isolates. Given the single-herd design and limited sample size, these findings should be considered descriptive, farm-level observations and should not be extrapolated to the Akmola region or Kazakhstan as a whole.

The higher frequency of *S. aureus* isolation among the animals after cefquinome treatment is one of the important observations of this study. However, this result should not be interpreted as evidence of *S. aureus* selection under the influence of cefquinome or as an indicator of treatment failure. Due to the observational design of the study, treatments were prescribed by the resident farm veterinarians, and pre-treatment samples were unavailable. Therefore, the result should be correctly considered as a characteristic of the bacterial composition of samples obtained 48 h after the end of treatment. Such a cautious interpretation is especially important for *S. aureus*, since this pathogen can form persistent intra-udder infections [29] and often exhibits lower bacteriological cure rates than many other mastitis pathogens [30,31].

Streptococci were detected in both animals treated with cefquinome and those with no previous antibiotic treatment and comprised two clinically significant mastitis-associated species, *Streptococcus dysgalactiae* and *Streptococcus uberis*. *S. uberis* is primarily classified as an environmental mastitis pathogen; however, its epidemiology is not restricted to environmental transmission; the possibility of transmission between animals has been demonstrated for individual clonal lines [32,33]. Consistent with this, all six *S. uberis* isolates in the present study carrying the identical *mef(J)–msr(I)–spw* resistance-gene cluster shared an identical MLST allelic profile, which did not correspond to a currently defined sequence type in the PubMLST database. This finding is most consistent with clonal relatedness among these isolates, rather than independent horizontal acquisition of a mobile element across genetically distinct strains and lends further support to the potential role of clonal spread within this herd; distinguishing clonal spread from mobile-element-mediated horizontal transfer with greater confidence would require additional strain-level resolution, such as core-genome SNP analysis. Likewise, *S. dysgalactiae* cannot be assigned exclusively to a single epidemiological category, as it may originate from both contagious and environmental sources [34]. Consequently, the detection of these two species reflects the mixed bacteriological profile of the herd but does not permit identification of the predominant transmission route [35]. At the same time, *S. uberis* remains one of the main causative agents of clinical and subclinical mastitis [36], particularly in herds where environmental reservoirs continue to play a major role despite effective control of classical contagious pathogens [37].

Non-*aureus* staphylococci (NAS) identified in the present study included *S. chromogenes*, *S. hyicus*, *S. simulans*, *S. epidermidis*, and *S. haemolyticus*. The most frequently detected non-*aureus* staphylococcal species were *S. chromogenes* and *S. hyicus*. This species distribution is consistent with the current understanding of NAS as a heterogeneous group of staphylococci whose species differ in their epidemiology, pathogenicity, impact on udder health, and antimicrobial resistance profiles [38]. In published studies, *S. chromogenes* is among the most frequently detected NAS in intra-udder infections and may be associated with increased somatic cell counts in milk [39,40]. Therefore, the detection of multiple NAS species in this study highlights the importance of species-level identification in the bacteriological diagnosis of mastitis, as grouping all NAS isolates together may obscure important differences in their clinical relevance, epidemiology, and antimicrobial resistance [41]. At the same time, because NAS may also originate from teat skin or sample contamination, their isolation, particularly as part of mixed cultures, should be interpreted cautiously.

Mixed cultures were detected in 24.4% of cow samples (10 of 41). Their presence reflects the complexity of the bacteriological picture of clinical mastitis and emphasizes the need to isolate and identify each morphologically distinct isolate. The practical significance of mixed cultures is that bacteria isolated from a single sample may differ in biology, source of infection, and antimicrobial susceptibility. Therefore, bacteriological diagnostics for mastitis should aim not only to identify the primary pathogen but also to correctly interpret polymicrobial results [42]. However, quantitative colony counts and semi-quantitative growth categories were not recorded in the present study, and no CFU-based threshold was applied. Therefore, contamination cannot be completely excluded, particularly for NAS, *Corynebacterium stationis*, and other organisms that may also originate from teat skin or the sampling environment.

Phenotypic testing revealed that tetracycline resistance was the most common AMR phenotype, detected in 71.7% (33 of 46) of isolates tested against this agent. It was particularly frequent among *S. uberis* (8/8, 100%), *S. dysgalactiae* (7/8, 87.5%), *S. aureus* (8/11, 72.7%), and *S. chromogenes* (5/7, 71.4%). This result is consistent with data from Northern Kazakhstan, where pronounced resistance to tetracyclines and β-lactams was also described in mastitis isolates of *S. aureus* [43,44], as well as with data on *S. uberis* from the Czech Republic, where tetracycline resistance was the most frequent phenotype and accounted for 59% [45]. Thus, tetracycline resistance was the predominant resistance phenotype in the studied bacterial collection. In contrast to tetracyclines, resistance to most β-lactams, aminoglycosides, fluoroquinolones, and trimethoprim/sulfamethoxazole was detected less frequently. Resistance to ampicillin was detected in 4/27 (14.8%) isolates, erythromycin in 6/45 (13.3%), trimethoprim/sulfamethoxazole in 4/40 (10.0%), ceftriaxone in 1/11 (9.1%), ciprofloxacin in 1/12 (8.3%), penicillin in 1/28 (3.6%), cefotaxime in 1/16 (6.2%), and imipenem in 2/40 (5.0%). Resistance to gentamicin and cefoxitin was detected in only one isolate each (1/40, 2.5%). Resistance of one *S. uberis* isolate to several β-lactams warrants cautious interpretation, as published data for this species more often describe retained susceptibility to β-lactams or intermediate-category β-lactam activity rather than resistance [46,47].

The MDR phenotype was detected in 6.9% (4 of 58) isolates: *S. chromogenes* P71-C8, *S. epidermidis* P71-E8, *S. uberis* P71-H6, and *Corynebacterium stationis* P71-C1. Isolate P71-H6 (*S. uberis*) showed the broadest resistance phenotype, with resistance to penicillin, ampicillin, cefotaxime, erythromycin, clindamycin, and tetracycline. This figure should be considered as a characteristic of the studied bacterial collection and not as an estimate of the prevalence of MDR in the herd, the Akmola region, or Kazakhstan. According to Magiorakos et al., MDR is defined as acquired non-susceptibility to at least one agent in three or more antimicrobial categories [48]. However, applying this definition to non-*aureus* staphylococci, streptococci, and other Gram-positive species is an operational approximation, since the original MDR/XDR/PDR scheme was developed primarily for epidemiologically important bacterial groups with standardized antimicrobial categories. Therefore, the interpretation of MDR in the present study should be made with caution, given the small sample size, species heterogeneity, differences in antimicrobial susceptibility testing panels, and the limited availability of species-specific interpretive criteria.

WGS analysis revealed that tetracycline resistance is the dominant feature of this isolate collection, present in 26 of 33 isolates (78.8%) via a mix of ribosomal protection genes (*tet(M)*, *tet(O)*) and efflux pumps (*tet(L)*, *tet(K)*, *tet(C)*, *tet(38)*)—consistent with sustained historical tetracycline use in dairy production and suggesting limited clinical utility of this class against the sampled population.

A distinct multidrug-resistant subgroup emerged within *Streptococcus uberis*: six isolates each carried a near-identical seven-to-eight-gene cassette spanning tetracycline, lincosamide/streptogramin A (*lnu(B)*, *lsa(E)*), macrolide (*mef(J)*, *msr(I)*), and aminoglycoside (*ant(6)-Ia*, *spw*) resistance. As noted above, these six isolates shared an identical MLST allelic profile, indicating that this consistent gene cassette most likely reflects clonal spread of a single strain within the herd, rather than independent horizontal acquisition of a shared mobile element across unrelated strains. This finding is clinically significant regardless of its underlying mechanism, since lincosamides (e.g., pirlimycin) and macrolides are first-line intramammary treatments for streptococcal mastitis, and the presence of this resistance cassette in a single circulating clone suggests that its spread within the herd could be curtailed through case-level infection control rather than requiring broader antimicrobial stewardship measures aimed at limiting horizontal gene transfer.

The single *Staphylococcus epidermidis* isolate carried the most diverse staphylococcal profile, including an intact *mecA* methicillin-resistance genotype with its regulator *mecR1*, the penicillinase gene *blaZ* with regulator *blaI*, plus *tet(K)*, *catA*, and *fosB*—notable because coagulase-negative staphylococci can act as reservoirs of mobile methicillin-resistance elements. *Corynebacterium stationis* and two *S. dysgalactiae* isolates carried the next-most diverse profiles, adding hygromycin (*aph(4)-Ia*), biocide/disinfectant (*qacE*, *qacEΔ1*), streptothricin (*sat4*), sulfonamide (*sul1*), and trimethoprim (*dfrA1*) resistance not seen elsewhere in the collection.

By contrast, roughly half of isolates (17/33) carried either no acquired resistance genes (6 isolates) or only a single tetracycline gene (11 isolates), indicating the multidrug profiles above are concentrated in a minority of strains rather than typical of the collection.

PlasmidFinder further revealed that plasmid replicons, predominantly of the staphylococcal repUS43 and repUS76 families, were present in the majority of screened isolates (21/33) (Supplementary Table S4). This finding indicates a potential role of plasmids in the dissemination of resistance determinants within this herd; however, direct physical linkage between the detected AMR genes and these plasmid replicons was not established in this study, and plasmid-mediated transmission was not directly demonstrated.

The present study has several limitations. The relatively small sample size and inclusion of cows from a single commercial dairy herd limit the generalizability of the findings. The observational design, absence of randomization, and lack of pre-treatment samples also preclude causal interpretation of differences between animals with and without prior cefquinome treatment. In addition, quantitative colony counts and semi-quantitative growth categories were not recorded, limiting the ability to distinguish mastitis-associated isolates from possible contaminants in some cases. Therefore, the bacterial species distribution and antimicrobial resistance patterns described here should be regarded strictly as descriptive, farm-level observations and hypothesis-generating findings rather than as estimates representative of the Akmola region or Kazakhstan. Larger multi-herd studies are required to determine whether the patterns observed in this herd are representative of broader dairy cattle populations.

## 4. Materials and Methods

### 4.1. Study Design and Herd Background

The study was observational and comparative in nature and was conducted on a commercial dairy farm in the Akmola region. The analysis included Simmental cows with clinical signs of mastitis for which veterinary records documented the use of antibacterial drugs, their administration schedules, and sampling time. Animals with incomplete or conflicting data on prior antimicrobial therapy were excluded, and a total of 41 cows were included in the analysis. Milk samples were collected by farm veterinarians during routine herd health management and diagnostic procedures. No additional animal handling or sampling was performed for research purposes.

The animals were divided into two groups based on their previous treatment. The first group consisted of 15 cows that were given cefquinome (Cobactan^®^ 2.5%, Intervet International B.V., Boxmeer, The Netherlands) at a dose of 1 mg/kg body weight (2 mL per 50 kg) once a day, for two consecutive days; milk samples were collected 48 h after the last administration. The second group consisted of 26 cows that had not received any antibacterial treatment prior to sampling during the current mastitis episode.

Antibacterial therapy was prescribed by the farm’s veterinarians as part of routine treatment and was not determined by this study’s protocol. Due to the lack of randomization and pretreatment samples, the study was not designed to evaluate the clinical or bacteriological efficacy of cefquinome. The term “control group” is not used; the groups are designated as “after cefquinome treatment” and “without prior antibiotic therapy”.

### 4.2. Sample Collection

Milk samples from cows with clinical signs of mastitis were collected using aseptic techniques. Prior to sample collection, udders and teats were cleared of visible debris and dried using individual disposable wipes. Teats were then disinfected with a 0.5% chlorhexidine solution for 30 s and dried with a clean, single-use towel. Immediately before collection, the teat tip was additionally wiped with 70% ethyl alcohol. The first three streams of milk were milked and discarded, after which the sample was collected in sterile disposable 15 mL tubes, preventing the tube and its cap from coming into contact with the teat or surrounding surfaces. If multiple udder quarters were affected, the sample was collected from a single, proximal quarter. The collected samples were placed in a thermal container with cooling elements, transported to the laboratory at 2–4 °C, and inoculated within 12 h of collection.

### 4.3. Bacteriological Culture and Isolation

Bacteriological culture of milk samples was performed using selective and differential solid media, including Endo agar (HiMedia Laboratories Pvt. Ltd., Mumbai, India), CHROMagar *Staphylococcus aureus* (CHROMagar, Paris, France), and Columbia agar supplemented with 5% defibrinated horse blood (Condalab, Madrid, Spain).

All media were prepared and used in accordance with the manufacturers’ instructions. Milk samples were inoculated onto the culture media under aseptic conditions and incubated aerobically at 37 °C for 24–48 h.

After incubation, bacterial growth was assessed by colony morphology, hemolytic activity, and cultural purity. Colonies with distinct morphological characteristics were subcultured to obtain pure isolates for subsequent species identification and downstream analyses.

### 4.4. Bacterial Identification by MALDI-TOF MS

Species-level identification of bacterial isolates was performed using matrix-assisted laser desorption/ionization time-of-flight mass spectrometry (MALDI-TOF MS) on a Microflex LT instrument (Bruker Daltonics, Bremen, Germany). For sample preparation, individual colonies were applied onto the target plate and overlaid with 1 μL of saturated α-cyano-4-hydroxycinnamic acid (α-HCCA) matrix solution containing 50% acetonitrile and 2.5% trifluoroacetic acid (TFA), followed by air drying at room temperature. Mass spectra were acquired in automatic mode using the manufacturer’s standard settings. The analyzed mass-to-charge ratio (*m*/*z*) range was 2000–20,000 Da. Spectral analysis and taxonomic assignment were performed using Bruker MALDI Biotyper software version 4.0. A score value of ≥2.0 was considered acceptable for reliable taxonomic assignment.

### 4.5. Antimicrobial Susceptibility Testing

Antimicrobial susceptibility testing was performed for all recovered bacterial isolates using the disk diffusion method in accordance with the recommendations of the European Committee on Antimicrobial Susceptibility Testing (EUCAST, version 16.0, 2026, available from https://www.eucast.org). Bacterial suspensions were prepared from fresh pure cultures and adjusted to a turbidity equivalent to a 0.5 McFarland standard. For *Staphylococcus* spp. and *Escherichia coli*, bacterial suspensions were inoculated onto Mueller–Hinton agar (TM Media, Titan Biotech Ltd., Bhiwadi, India) using a sterile cotton swab to obtain a uniform bacterial lawn. Plates were incubated aerobically at 35 ± 1 °C for 18 ± 2 h, and inhibition zone diameters were interpreted according to the EUCAST clinical breakpoints established for *Staphylococcus* spp. and Enterobacterales, respectively. For *Streptococcus* spp. and *Corynebacterium stationis*, bacterial suspensions were inoculated onto Mueller–Hinton agar supplemented with 5% defibrinated horse blood and 20 mg/L β -nicotinamide adenine dinucleotide (MH-F medium). Plates were incubated at 35 ± 1 °C in an atmosphere containing 5% CO_2_ for 18 ± 2 h. Inhibition zone diameters were interpreted according to the EUCAST clinical breakpoints for streptococci and *Corynebacterium* spp. other than *Corynebacterium diphtheriae* and *Corynebacterium ulcerans*, respectively. Commercial antimicrobial disks (Research Center of Pharmacotherapy, Saint Petersburg, Russia) were applied to the agar surface within 15 min after inoculation. The following incubation, inhibition zone diameters were measured and interpreted according to the corresponding EUCAST breakpoint tables. Species-specific antimicrobial panels were used, and only antimicrobial agents with available EUCAST interpretive criteria for the respective bacterial groups were included in the final analysis.

### 4.6. DNA Extraction

Genomic DNA was extracted from pure bacterial cultures using the QIAamp DNA Mini Kit (Cat. no. 51306, QIAGEN, Hilden, Germany) according to the manufacturer’s protocol. The concentration and purity of the extracted DNA were assessed using a Qubit double-stranded DNA HS Assay Kit (Thermo Fisher Scientific, Waltham, MA, USA).

### 4.7. Whole Genome Sequencing Analysis

A total of 58 bacterial isolates were available for the study. A subset of 33 isolates was selected for downstream analysis using simple random sampling without replacement. Random selection was performed using the sample() function in the R statistical environment. Accordingly, each of the 58 isolates had an equal probability of being included in the sample, and the final sample comprised 33 unique isolates. All WGS-derived percentages reported in this study refer specifically to this sequenced subset of 33 isolates and should not be extrapolated to the full 58-isolate collection.

DNA libraries were prepared using the MGIEasy FS PCR-Free DNA Library Prep Kit V1.2 (Cat. No. 1000013455, MGI, Shenzhen, China). Sequencing was performed on the DNBSEQ-G50RS platform (MGI, Shenzhen, China) using the reagent cartridge and flow cell from the DNBSEQ-G50RS High-throughput Sequencing Set (FCL PE100) (Cat. No. 940-001634-00, MGI, Shenzhen, China).

### 4.8. Genome Assembly and Bioinformatics Analysis

The quality of raw reads obtained in the FASTQ format was assessed using FastQC (v0.12.1, Babraham Bioinformatics) [49]. The reports obtained for each sample were combined and visualized using MultiQC (v1.35) [50] for comparative quality analysis of all samples.

Adapter trimming and filtering of low-quality paired-end reads were performed using fastp (v1.3.6) [51]. Adapter sequences were detected automatically (--detect_adapter_for_pe), low-quality bases were trimmed from the 5′- and 3′-ends of reads (--cut_front, --cut_tail) at a quality threshold of Phred Q ≥ 20 (--qualified_quality_phred 20); reads shorter than 50 bp after processing were excluded from analysis (--length_required 50). HTML and JSON reports were generated for each sample.

De novo genome assembly for each sample was performed using the SPAdes assembler (v4.3.0) [52] in isolate mode (--isolate), designed for assembling bacterial genomes obtained from high-coverage pure cultures. The resulting contigs (contigs.fasta) were extracted and renamed according to their sample IDs.

The quality and statistical characteristics of the resulting genome assemblies (number of contigs, N50, total length, GC composition, etc.) were assessed using the QUAST (v5.3.0) program [53].

Functional annotation of the assembled genomes was performed using the Prokka (v1.15.6) program [54]. To predict protein-coding sequences and rRNA/tRNA genes, the --centre X and --compliant parameters were used to align contig names with NCBI (GenBank) requirements.

A search for acquired antimicrobial resistance genes, virulence factors, and plasmid replicons was conducted using several independent tools and databases: AMRFinderPlus (v3.11.14, database version 2026-01-20.1, NCBI), Abricate v1.4.0 using ResFinder database for resistance genes, and PlasmidFinder for plasmids detection. Multilocus sequence typing (MLST) of the assembled genomes was performed using the mlst program (v2.32.3, T. Seemann), which uses PubMLST database typing schemes, to determine the sequence types (STs) of the studied strains.

### 4.9. Definition of Multidrug Resistance

Multidrug resistance (MDR) was defined according to the criteria proposed by Magiorakos et al. [48], whereby an isolate was considered multidrug-resistant if it exhibited resistance to three or more antimicrobial classes. For this study, MDR classification was applied to isolates with available phenotypic antimicrobial susceptibility testing data.

### 4.10. Descriptive Data Analysis

Data were summarized using descriptive statistics. The isolation frequencies of bacterial species, antimicrobial resistance (AMR) phenotypes, and AMR genes were expressed as absolute counts and percentages. Due to the descriptive design of this study, no inferential statistical analyses were performed.

## 5. Conclusions

In this single studied Simmental herd in Kazakhstan, clinical mastitis was predominantly associated with staphylococci, with *Staphylococcus aureus* and *Escherichia coli* being the most frequently isolated pathogens (*n* = 11 each), along with significant proportions of *Streptococcus* spp. Tetracycline resistance emerged as the most pronounced antimicrobial resistance (AMR) trait at both the phenotypic and genomic level, detected in the majority of Gram-positive isolates by disk diffusion and confirmed by the presence of tetracycline resistance genes in 26 of 33 sequenced isolates, while multidrug-resistant (MDR) phenotypes and genotypes were each limited to a small subset of strains. Whole-genome sequencing substantially extended the resolution of AMR characterization beyond phenotypic testing and targeted molecular screening: AMRFinderPlus and ResFinder identified acquired resistance genes in 27 of 33 isolates (82%), spanning tetracycline, lincosamide/macrolide, and aminoglycoside determinants, with AMRFinderPlus consistently detecting an equal or broader gene repertoire than ResFinder. PlasmidFinder further revealed that plasmid replicons, predominantly of the staphylococcal repUS43 and repUS76 families, were present in the majority of screened isolates (21/33), suggesting a potential role of plasmids in the dissemination of resistance determinants within this herd, although direct linkage between the detected AMR genes and these plasmids was not established. Taken together, these genomic findings corroborate and considerably expand upon the phenotypic and targeted molecular screening results, underscoring that comprehensive AMR surveillance benefits from combining phenotypic antibiograms with whole-genome sequencing rather than relying on either approach alone. As this study was limited to a single herd, these findings should be regarded as descriptive, farm-level observations rather than being representative of Kazakhstan as a whole; nonetheless, they highlight the potential value of localized mastitis monitoring programs integrating accurate species identification, routine phenotypic antibiograms, and genomic surveillance of resistance genes and their mobile genetic elements.

## Figures and Tables

**Table 1:** Species composition of 58 bacterial isolates recovered from 41 milk samples collected from cows with clinical mastitis, depending on previous antibiotic therapy.

Bacterial Species	Total Isolates ^1^, *n* = 58	Treated Group (15 Cows), Isolates *n* = 32	Untreated Group (26 Cows), Isolates *n* = 26
*Staphylococcus aureus*	11	11	0
*Staphylococcus chromogenes*	7	7	0
*Staphylococcus hyicus*	7	7	0
*Staphylococcus simulans*	2	2	0
*Staphylococcus epidermidis*	1	1	0
*Staphylococcus haemolyticus*	1	1	0
*Corynebacterium stationis*	1	0	1
*Escherichia coli*	11	3	8
*Streptococcus dysgalactiae*	8	0	8
*Streptococcus uberis*	8	0	8
*Aerococcus viridans*	1	0	1

^1^ Data represent the number (*n*) of bacterial isolates from which each corresponding species was isolated. Because multiple species were recovered from some samples, the sum of isolates across species (*n* = 58) exceeds the number of milk samples examined (*n* = 41).

**Table 2:** AMR gene calls identified using AMRFinderPlus among 33 isolates.

Gene	Isolates Affected	Associated Phenotype/Function
*mef(J)*	6	Macrolide efflux transporter
*msr(I)*	6	ABC-F macrolide/lincosamide/streptogramin protection
*spw*	6	ANT(9)-family aminoglycoside (spectinomycin) resistance
*tet(38)*	4	Staphylococcal tetracycline efflux pump (chromosomal)
*erm(B)*	3	MLS_B_ ribosomal methylase
*aadE*	2	Aminoglycoside (streptomycin) adenylyltransferase
*sat4*	2	Streptothricin acetyltransferase
*tet(L)*	2	Tetracycline efflux transporter
*erm(56)*, *blaI*, *fosB*, *mecR1*, *qacE*, *qacEΔ1*	1 each	MLS_B_ methylase; *mecA*/*blaZ* regulatory repressors; fosfomycin thiol-transferase; quaternary-ammonium/biocide efflux

## Data Availability

The sequencing data generated in this study have been deposited in the NCBI Sequence Read Archive (SRA) under BioProject accession number PRJNA1523028, with individual BioSample accession numbers SAMN62925384–SAMN62925416 (33 accessions, corresponding to the 33 sequenced isolates).

## Supplementary Materials

The following supporting information can be downloaded at https://www.mdpi.com/article/10.3390/antibiotics15090889/s1. Supplementary Table S1. Disk diffusion antimicrobial susceptibility results for bacterial isolates recovered from milk samples of cows with clinical mastitis. Supplementary Table S2. Antimicrobial resistance genes, gene presence/absence profiles, and multilocus sequence types (MLST) identified by whole-genome sequencing in 33 mastitis-associated bacterial isolates. Supplementary Table S3. Antimicrobial resistance genes identified by whole-genome sequencing in a subset of mastitis-associated bacterial isolates, as detected using AMRFinderPlus and ResFinder. Supplementary Table S4. Plasmid replicons identified by whole-genome sequencing (PlasmidFinder) in mastitis-associated bacterial isolates.

## Abbreviations

The following abbreviations are used in this manuscript:

NAS	Non-*aureus* staphylococci
MDR	Multidrug resistance
ARGs	Antimicrobial resistance genes
MGEs	Mobile genetic elements
